# Does Cardiovascular Mortality Overtake Cancer Mortality During Cancer Survivorship? An English Retrospective Cohort Study

**DOI:** 10.1016/j.jaccao.2022.01.102

**Published:** 2022-03-15

**Authors:** Helen Strongman, Sarah Gadd, Anthony A. Matthews, Kathryn E. Mansfield, Susannah Stanway, Alexander R. Lyon, Isabel dos-Santos-Silva, Liam Smeeth, Krishnan Bhaskaran

**Affiliations:** a Department of Non-Communicable Disease Epidemiology, London School of Hygiene and Tropical Medicine, London, United Kingdom; b Institute of Environmental Medicine, Karolinska Institute, Stockholm, Sweden; c Breast Unit, Royal Marsden Hospital, London, United Kingdom; d Royal Brompton Hospital and National Heart and Lung Institute, Imperial College London, London, United Kingdom; e HDR UK London, London, United Kingdom

**Keywords:** beyond cancer, cancer survivors, electronic health records, CPRD GOLD, Clinical Practice Research Datalink primary care data in England

## Abstract

**Background:**

Cancer survivors have a higher risk for developing cardiovascular diseases than the general population.

**Objectives:**

The aim of this study was to investigate whether cardiovascular mortality overtakes cancer-specific mortality during cancer survivorship and, if so, at what point cardiovascular disease becomes the dominant cause of death.

**Methods:**

This cohort study used linked English electronic health records, including death registration data. The study population included 104,028 adults ≥40 years of age whose first cancer diagnosis was for 1 of 9 common cancers and who were alive and followed up at least 1 year after diagnosis. Age-stratified mortality rates were estimated from cardiovascular disease or cancer by predicting from Poisson models incorporating categorical age at diagnosis and time since diagnosis. Where cardiovascular disease mortality overtook cancer mortality, the crossover point was estimated using interpolation.

**Results:**

Mortality from cardiovascular causes overtook mortality due to the primary cancer at 2 to 11 years after cancer diagnosis in survivors of all 9 cancer types ≥80 years of age at diagnosis and after 5 to 17 years in survivors of 7 cancer types 60 to 79 years of age at diagnosis. Cardiovascular mortality overtook all cancer mortality for 6 and 2 cancer sites in the ≥80-year and 60- to 79-year age groups, respectively, over a longer time period. Cardiovascular mortality did not overtake cancer mortality during the observation period in patients aged 40 to 59 years, except among survivors of uterine cancer.

**Conclusions:**

In older survivors of 9 common cancers, cardiovascular mortality becomes dominant over mortality from the primary cancer, though not always over total cancer mortality, as time passes since cancer diagnosis.

Cancer and cardiovascular disease are competing causes of death in England; each accounted for approximately 30% of deaths in 2019, with cardiovascular disease causing more deaths in older age groups (>70 years for women, >80 years for men) and neoplasms being dominant in younger age groups.^1^

With improvements in cancer detection and care, people are living longer after cancer.^2^ Adult survivors of most cancer sites are at higher risk for cardiovascular disease compared with the general population, with variation in the size and duration of risk among cancer sites and age groups.^3^ Compared with the general population, cardiovascular mortality has been shown to be elevated in survivors of cancer from several sites,^4^, ^5^, ^6^, ^7^ with most studies investigating breast cancer^8^ or lymphoma.^9^ To raise awareness of the risk for cardiovascular disease in cancer survivors and inform patient counseling and decisions about monitoring and priorities for disease prevention, it is important to establish how risk for death of cardiovascular disease competes with risk for death of cancer over time.

Studies from the United States have estimated that in people who survive breast and endometrial cancers, cardiovascular mortality exceeds mortality from the primary cancer after 10 to 15 and 5 years, respectively.^10^, ^11^, ^12^, ^13^ These analyses support long-term monitoring of cardiovascular risk in addition to cancer recurrence in breast and endometrial cancer survivors. It is not clear, however, how mortality risks from cancer and cardiovascular diseases compare over time following diagnosis of a wider range of cancers or how differences in these cause-specific mortality risks vary by age at cancer diagnosis.

We therefore aimed to identify whether, and at what time point, risk for cardiovascular mortality overtakes risk for mortality from the primary cancer and all cancers combined, by age, in survivors of the 9 most common cancers in England.

## Methods

For this retrospective cohort study, we identified cohorts of survivors of 9 different types of cancer using Clinical Practice Research Datalink primary care data in England (CPRD GOLD),^14^ linked to national data^15^ on hospital admissions from Hospital Episode Statistics Admitted Patient Care,^16^ cancer registrations from the National Cancer Registration and Analysis Service,^17^ and death registrations from the Office of National Statistics. CPRD GOLD data comprise anonymized electronic health records prospectively collected by primary care practices in the United Kingdom as part of routine clinical care. The dataset covers approximately 7% of the U.K. population and includes Read-coded diagnoses, prescriptions, clinical measurements, and test results. Linked datasets add International Classification of Diseases–coded cancer registrations, hospitalizations, and dates and causes of death. The study period (covered by all linked data sources) was January 1990 to December 2015.

This study was approved by the London School of Hygiene and Tropical Medicine ethics committee (12042) and the Independent Scientific Advisory Committee for the Medicines and Healthcare Products Regulatory Agency database research (16_274). CPRD supplies anonymized data for public health research; therefore individual patient consent was not required for this study.^15^

### Participants, exposures and outcomes

**Figure 1:**
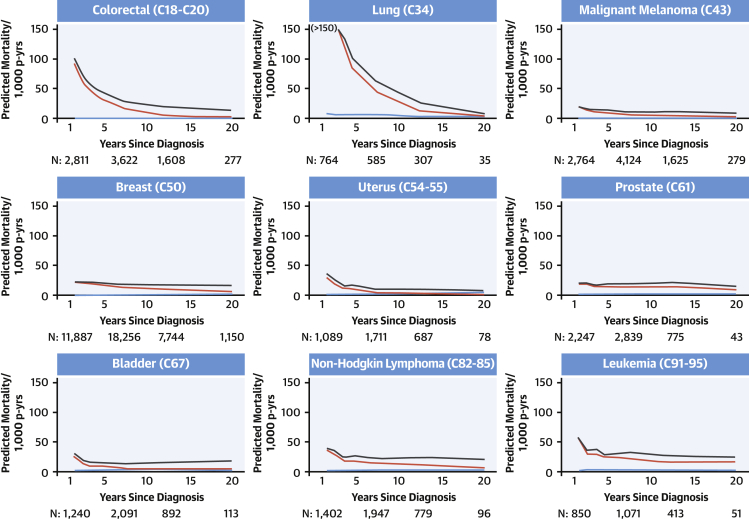
Predicted Cause-Specific Mortality in Cancer Survivors 40 to 59 Years of Age at Diagnosis Predicted cause-specific mortality per 1,000 person-years (p-yrs) by time since diagnosis in cancer survivors 40 to 59 years of age at diagnosis. Predicted mortality was calculated using Poisson models incorporating categorical age at diagnosis and time since diagnosis. Cardiovascular mortality did not overtake cancer mortality during the observation period, except among survivors of uterine cancer. **Blue line** = cardiovascular disease mortality; **red line** = primary malignancy mortality; **black line** = all malignancy mortality. Ordering of cancer sites is by International Classification of Diseases code of the cancer site. Predicted mortality is predicted at the midpoint of each category of time since diagnosis. The number of people (N) contributing to the analysis at 1.5, 7.5, 12.5, and 20 years is provided. Graphs are truncated at the earliest time since diagnosis group where the width of the CI exceeds 50 to 1,000 patient-years.

Cohort development has been described previously.^3^ We included individuals with a first ever cancer record from 1 of the 9 most common cancer sites (Figure 1) in Read-coded CPRD GOLD data or International Classification of Diseases-10th Revision–coded Hospital Episode Statistics Admitted Patient Care or National Cancer Registration and Analysis Service cancer registration data (cancer code lists are available at https://doi.org/10.17037/DATA.00001113). At least 1 year of research-quality^14^ follow-up was required prior to the first record of cancer, as determined by patient registration data and quality checks performed by CPRD,^14^ to ensure that these records reflected incident diagnoses. We included individuals alive and under CPRD follow-up 1 year after cancer diagnosis. Individuals were grouped into approximately 20-year age categories (40-59, 60-79, and ≥80 years). For this study, we excluded adults younger than 40 years from the cohort, because of small numbers of cardiovascular deaths in this age group.

Outcomes were deaths with underlying causes of cardiovascular disease (International Classification of Diseases-10th Revision chapter IX/International Classification of Diseases-9th Revision 390-459), the primary cancer, or any cancer (including the primary cancer), identified using linked death registration data. In a sensitivity analysis, we used the immediate rather than underlying cause of death.

### Statistical analysis

Follow-up began 1 year after cancer diagnosis (as the original study cohort was generated to examine disease risks during medium- to long-term survivorship rather than in the initial intense treatment period)^3^ and ended at the time of all-cause death or end of study period (December 31, 2015), whichever came first. By ending follow-up up at all-cause death, follow-up was censored at the competing risk for death by other causes in each model. This is a cause-specific hazard modeling approach^18^ and means that at each time point since diagnosis, our analysis includes cancer survivors who were alive at that time point; we believed that this would make our findings most relevant to current cancer survivors at different stages of survivorship. This differs from subdistribution hazard modeling approaches, which are commonly used to estimate clinical prognosis at the start of follow-up.^19^ We did not end follow-up when individuals left the primary care practice, as the outcome (cause-specific mortality) was measured in linked national death registration data. We described the number of individuals in the study population and for each age and cancer site grouping with the number and percentage who were female, median (IQR) follow-up from index, and the number of deaths due to cardiovascular disease, the primary malignancy, and all malignancies. We additionally described mean age at diagnosis for the full study population. Individual follow-up time was divided into groups of 1 to <2, 2 to <3, 3 to <4, 4 to <5, 5 to <10, 10 to <15, and 15 to 25 years since diagnosis. We fit Poisson models for each cancer site and cause-specific mortality outcome, with age and time since diagnosis groups as dependent categorical variables. We used these models to predict age-stratified cause-specific mortality rates per 1,000 person-years by years since diagnosis, which were then plotted against the midpoint of each time since diagnosis group (eg, 10 to <15 years plotted at 12.5 years). Where cardiovascular disease mortality overtook cancer mortality, the crossover point was estimated by interpolating between points on either side of the crossover point; 95% CIs were estimated by taking the 2.5th and 97.5th percentile estimates from 200 bootstrap repetitions. In secondary analyses, we repeated these analyses separately for men and women. Statistical analyses were done in Stata MP version 16 (StataCorp).

## Results

Of the 126,120 cancer survivors identified previously,^3^ 107,281 individuals were diagnosed with 1 of the 9 most common cancers, and 104,028 were aged at least 40 years at diagnosis. Mean age was 67.7 ± 11.9 years and 52,713 cancer survivors (50.7%) were women. We observed 7,091 cardiovascular deaths and 25,666 cancer deaths, of which 19,758 were due to the primary cancer, over a median follow-up time from 1 year after cancer diagnosis of 4.8 years (IQR: 2.0-8.7 years). Table 1 describes study population denominators and outcomes by cancer site and age group.

**Table 1:** Study Population Denominators and Outcomes by Cancer Site and Age Group

Cancer site	Age (at Diagnosis), y	Individuals	Female	Years of Follow-Up From Index^a^	Deaths		
					Cardiovascular	Primary Malignancy	All Malignancies
All	67.7 ± 11.9	104,028	52,713 (50.7)	4.8 (2.0-8.7)	7,091	19,758	25,666
Bladder (C67)	40-59	1,288	324 (25.2)	7.7 (3.9-11.9)	32	99	172
	60-79	5,640	1,230 (21.8)	5.3 (2.4-9.1)	598	714	1,316
	≥80	2,080	594 (28.6)	3.1 (1.3-5.7)	367	384	600
Breast (C50)	40-59	12,208	12,208 (100.0)	7.1 (3.6-11.4)	88	1,486	1,713
	60-79	12,669	12,669 (100.0)	6.1 (3.0-9.9)	661	1,701	2,304
	≥80	3,793	3,793 (100.0)	3.2 (1.4-5.9)	646	879	1,064
Colorectal (C18-C20)	40-59	3,029	1,364 (45.0)	5.4 (2.0-10.1)	29	690	856
	60-79	10,029	4,125 (41.1)	4.6 (1.9-8.5)	638	2,189	3,057
	≥80	3,471	1,881 (54.2)	2.9 (1.0-5.8)	500	948	1,304
Leukemia (C91-C95)	40-59	903	344 (38.1)	5.8 (2.7-9.6)	16	168	198
	60-79	2,217	885 (39.9)	4.7 (2.0-8.0)	151	489	664
	≥80	759	371 (48.9)	2.6 (1.0-5.3)	114	177	234
Lung (C34)	40-59	1,044	510 (48.9)	1.5 (0.5-5.2)	25	593	631
	60-79	4,102	1,723 (42.0)	1.3 (0.5-3.9)	183	2,373	2,544
	≥80	1,016	504 (49.6)	0.9 (0.4-2.2)	93	607	646
Melanoma (C43)	40-59	2,850	1,673 (58.7)	6.9 (3.4-10.8)	12	200	256
	60-79	3,396	1,684 (49.6)	5.4 (2.5-9.2)	197	358	556
	≥80	1,030	599 (58.2)	3.1 (1.3-5.9)	158	122	229
NHL (C82-C85)	40-59	1,450	622 (42.9)	6.4 (3.0-10.6)	25	200	263
	60-79	2,674	1,293 (48.4)	4.8 (2.1-8.1)	203	535	718
	≥80	721	418 (58.0)	2.9 (1.3-5.1)	114	188	250
Prostate (C61)	40-59	2,341	0 (0.0)	5.8 (2.9-9.4)	29	229	264
	60-79	16,742	0 (0.0)	5.1 (2.5-8.5)	1,182	2,647	3,533
	≥80	4,677	0 (0.0)	2.9 (1.3-5.1)	818	1,271	1,560
Uterus (C54-C55)	40-59	1,133	1,133 (100.0)	7.2 (3.6-11.2)	18	88	128
	60-79	2,328	2,328 (100.0)	5.5 (2.4-9.2)	120	325	473
	≥80	438	438 (100.0)	3.5 (1.2-6.0)	74	98	133

Values are mean ± SD, n, n (%), or median (IQR).

NHL = non-Hodgkin lymphoma.

a 1 year following cancer diagnosis.

**Figure 2:**
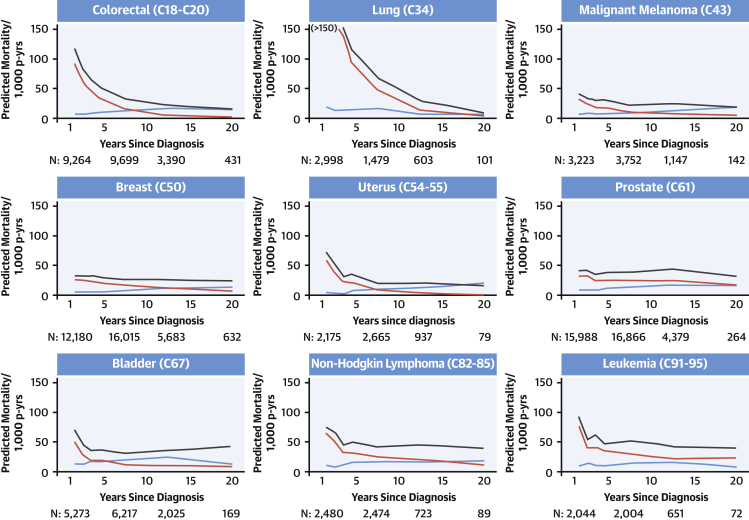
Predicted Cause-Specific Mortality in Cancer Survivors 60 to 79 Years of Age at Diagnosis Predicted cause-specific mortality per 1,000 person-years (p-yrs) by time since diagnosis in cancer survivors 60 to 79 years of age at diagnosis. Predicted mortality was calculated using Poisson models incorporating categorical age at diagnosis and time since diagnosis. Mortality from cardiovascular causes overtook mortality due to the primary cancer in survivors of 7 of 9 cancer types. Cardiovascular mortality overtook all cancer mortality for malignant melanoma and uterine cancer over a longer time period. **Blue line** = cardiovascular disease mortality; **red line** = primary malignancy mortality; **black line** = all malignancy mortality. Ordering of cancer sites is by International Classification of Diseases code of the cancer site. Predicted mortality is predicted at the midpoint of each category of time since diagnosis. The number of people (N) contributing to the analysis at 1.5, 7.5, 12.5, and 20 years is provided. Graphs are truncated at the earliest time since diagnosis group where the width of the CI exceeds 50 to 1,000 patient-years.

**Figure 3:**
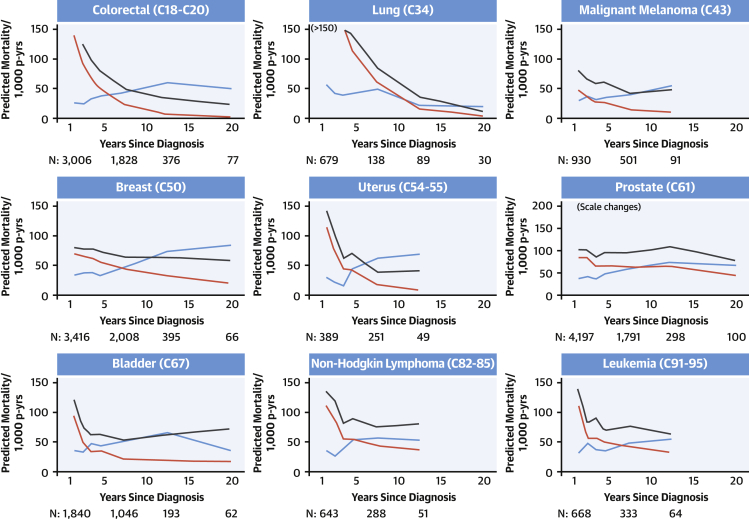
Predicted Cause-Specific Mortality in Cancer Survivors ≥80 Years of Age at Diagnosis Predicted cause-specific mortality per 1,000 person-years (p-yrs) by time since diagnosis in cancer survivors ≥80 years of age at diagnosis. Predicted mortality was calculated using Poisson models incorporating categorical age at diagnosis and time since diagnosis. Mortality from cardiovascular causes overtook mortality due to the primary cancer in survivors of all 9 cancer types. Cardiovascular mortality overtook all cancer mortality for 6 cancer types over a longer time period. **Blue line** = cardiovascular disease mortality; **red line** = primary malignancy mortality; **black line** = all malignancy mortality. Ordering of cancer sites is by International Classification of Diseases code of the cancer site. Predicted mortality is predicted at the midpoint of each category of time since diagnosis. The number of people (N) contributing to the analysis at 1.5, 7.5, 12.5, and 20 years is provided. Graphs are truncated at the earliest time since diagnosis group where the width of the CI exceeds 50 to 1,000 patient-years.

Mortality over time due to cardiovascular disease, the primary cancer, and all cancers by cancer site is shown in Figure 1, Figure 2, and Figure 3 for patients 40 to 59, 60 to 79, and ≥80 years of age at diagnosis, respectively. Mortality from the primary cancer, 1 year after diagnosis, was highest for people with primary lung and colorectal cancer; moderate for those with malignant melanoma, uterine cancer, bladder cancer, non-Hodgkin lymphoma, and leukemia; and lowest for those with breast and prostate cancer. For all cancer survivors, rate of mortality from the primary cancer declined over time. There was a clear pattern of increasing mortality from cardiovascular disease over time for older survivors of most of the 9 primary cancers examined, which was more pronounced in the ≥80-year age group than the 60- to 79-year age group. Cardiovascular mortality remained low throughout follow-up in cancer survivors 40 to 59 years of age at diagnosis.

Crossover points and 95% CIs where risk for cardiovascular mortality overtook risk for mortality due to the primary and all cancers are described in Table 2. Mortality due to cardiovascular disease overtook mortality due to the primary cancer for all cancers examined among survivors ≥80 years of age at diagnosis at time points ranging from 1.9 to 10.6 years after diagnosis and, for all cancers except leukemia and prostate cancer in the 60- to 79-year age group, at time points ranging from 4.8 to 17.4 years. The earliest crossover points for these age groups were for malignant melanoma, bladder cancer, and uterine cancer. In those 40 to 59 years of age at diagnosis, cardiovascular mortality remained low over time and overtook primary cancer mortality only among uterine cancer survivors (after 11.0 years; 95% CI: 8.2-20.8 years).

**Table 2:** Crossover Point at Which Cardiovascular Disease Mortality Rate Equals Cancer Mortality Rate

Cancer Site	Age Group (at Diagnosis)		
	40-59 Years	60-79 Years	≥80 Years
Primary malignancy			
Colorectal (C18-C20)		8.6 (8.1-13.9)	5.4 (4.7-9.3)
Lung (C34)		17.4 (12.9-26.6)	10.6 (7.4-22.4)
Malignant melanoma (C43)		8.5 (6.8-14.6)	1.9 (1.5-4.5)
Breast (C50)		12.7 (11.6-21.6)	7.1 (6.3-12.6)
Uterus (C54-C55)	11.0 (8.2-20.8)	7.1 (6.6-13.0)	3.9 (3.5-8.8)
Prostate (C61)			8.9 (7.4-16.7)
Bladder (C67)		4.8 (2.9-9.1)	2.6 (2.4-3.9)
Non-Hodgkin lymphoma (C82-C85)		15.4 (10.8-24.8)	4.7 (3.1-9.9)
Leukemia (C91-C95)			6.3 (2.9-13.1)
All malignancies			
Colorectal (C18-C20)			8.3 (7.0-13.2)
Lung (C34)			17.1 (11.5-26.2)
Malignant melanoma (C43)		19.6 (14.4-26.2)	8.8 (4.1-15.2)
Breast (C50)			10.4 (8.3-15.1)
Uterus (C54-C55)		17.5 (11.7-24.7)	5.9 (4.4-10.3)
Prostate (C61)			
Bladder (C67)			9.3 (3.9-14.1)
Non-Hodgkin lymphoma (C82-C85)			
Leukemia (C91-C95)			

Tabulated crossover point (years, 95% CI) for each cancer site and age stratum is the point at which the predicted cardiovascular disease mortality rate equals the predicted mortality rate from the cancer. Predicted cardiovascular and cancer mortality rates over time were calculated using Poisson models including categorical age at diagnosis and time since diagnosis, and crossover points were calculated by interpolating between the time points at either side of the intersection; 95% CIs were estimated by taking the 2.5th and 97.5th percentile estimates from 200 bootstrap repetitions. Crossover points are not provided if the cardiovascular mortality rate did not equal the mortality rate from cancer within 20 years.

Mortality due to cardiovascular mortality overtook mortality due to any cancer for survivors of 6 cancers in those ≥80 years of age at diagnosis at time points ranging from 5.9 to 17.1 years after diagnosis; the earliest crossover points were for uterine cancer, colorectal cancer, and malignant melanoma. In cancer survivors 60 to 79 years of age at diagnosis, cardiovascular mortality overtook mortality due to any cancer after 17.5 years (95% CI: 11.7-24.7 years) and 19.6 years (95% CI: 14.4-26.2 years) in survivors of uterine cancer and malignant melanoma, respectively. No crossover was observed in those 40 to 59 years of age at diagnosis.

Cause-specific mortality patterns were similar in men and women (Supplemental Figures 1A to 1F). Our results were similar in a sensitivity analysis using immediate rather than underlying cause of death (Supplemental Figures 2A to 2C).

## Discussion

### Key findings

In this assessment of cause-specific mortality in survivors of the 9 most common cancers in England, we found that for older site-specific cancer survivors, cardiovascular mortality frequently overtook mortality from the primary cancer, though not always total cancer mortality, in the years after cancer diagnosis (Central Illustration). Among cancer survivors ≥80 years of age at diagnosis, cardiovascular mortality overtook mortality from the primary malignancy for all 9 cancer sites between 2 and 11 years after diagnosis, and cardiovascular mortality overtook total cancer mortality for survivors of 6 cancer sites between 6 and 17 years after diagnosis. In younger age groups, cancer mortality was more likely to remain dominant, with cardiovascular mortality overtaking primary malignancy mortality during the observation period for only 6 cancer sites (colorectal, lung, malignant melanoma, breast, uterus, bladder, and non-Hodgkin lymphoma) in patients 60 to 79 years of age at diagnosis and for only 1 cancer site (uterine cancer) in those 40 to 59 years of age. Crossover points were earliest for malignant melanoma, bladder cancer, and uterine cancer in the older age groups.

**Central Illustration:**
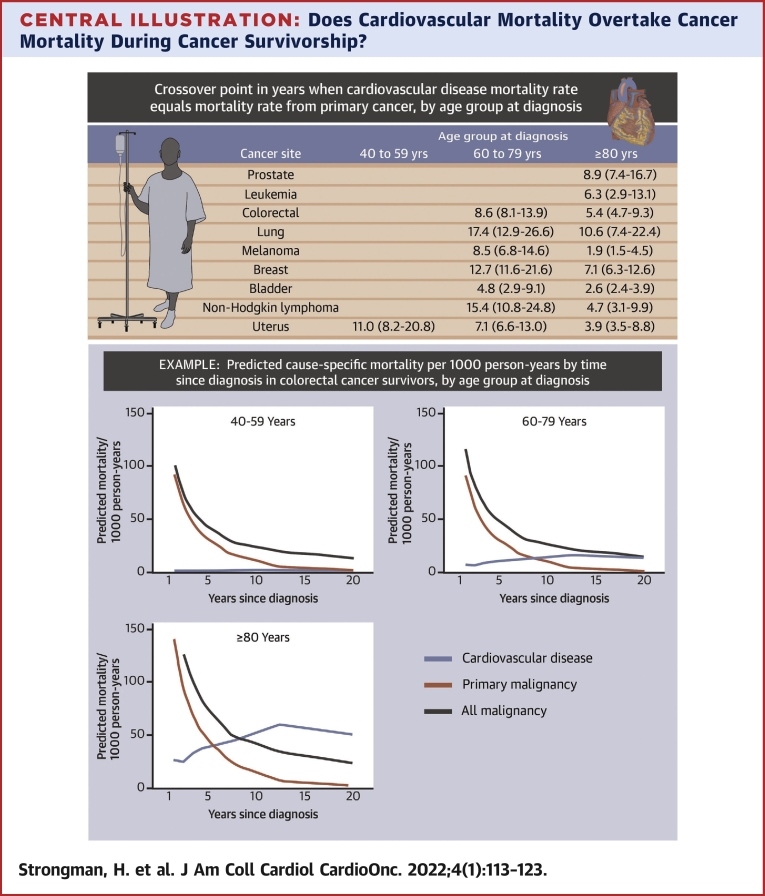
Does Cardiovascular Mortality Overtake Cancer Mortality During Cancer Survivorship? Tabulated crossover point (years, 95% CIs) at which the predicted cardiovascular disease mortality rate equals the predicted mortality rate from primary cancer for the 9 most common cancers, by age group at diagnosis. Predicted cardiovascular and cancer mortality rates over time were calculated using Poisson models including categorical age at diagnosis and time since diagnosis, and crossover points were calculated by interpolating between points at either side of the intersection; 95% CIs were estimated by taking the 2.5th and 97.5th percentile estimates from 200 bootstrap repetitions. Crossover points are not provided if the cardiovascular mortality rate did not equal the mortality rate from cancer within 20 years. An illustrative example is provided for colorectal cancer. In older survivors of 9 common cancers, cardiovascular mortality becomes dominant over mortality from the primary cancer as time passes since cancer diagnosis.

### Findings in relation to existing knowledge

To our knowledge, few prior studies with comparable data have been published. Higher cardiovascular mortality compared with breast cancer mortality was observed approximately 10 to 15 years following breast cancer diagnosis in women in the United States ≥66 years of age at death^10^ and a mean 61 years of age at breast cancer diagnosis, respectively.^13^ This is generally consistent with our study, in which the crossover points for breast cancer mortality were at 12.7 years (95% CI: 11.6-21.6 years) and 7.1 years (95% CI: 6.3-12.6 years) in people 60 to 79 and ≥80 years of age, respectively. Cardiovascular disease deaths were also previously reported to be more common than uterine cancer deaths in 5-year survivors in the United States,^11^^,^^12^ broadly corresponding to our estimated crossover points of 7.1 years (95% CI: 6.6-13.0 years) and 3.9 years (95% CI: 3.5-8.8 years) for uterine cancer survivors 60 to 79 and ≥80 years of age, respectively. Early cardiovascular deaths were more common in women with localized stage or low-grade tumors compared with women with advanced stage or aggressive histologic subtypes.^10^, ^11^, ^12^

Differences in crossover points between age groups appear to be driven primarily by different patterns of cardiovascular mortality over time. In the older age groups, we observed increases in risk for cardiovascular mortality over time since cancer diagnosis, often overtaking declining risk for primary and all cancer mortality. These clear increases in risk for cardiovascular diseases were not observed in cancer survivors 40 to 59 years of age. This is consistent with a previous analysis of data from this cohort in which absolute risk for incident fatal and nonfatal coronary artery disease, arrhythmia, heart failure or cardiomyopathy, and stroke in cancer survivors was highest in the oldest age groups, although relative risk was highest in younger people.^3^ In younger age groups, our findings are further supported by research in the United States describing a 1.4-fold relative increase in deaths in 5-year survivors of adolescent and young adult cancer survivors compared with the general population but low rates of excess cardiovascular deaths (3.6 per 10,000 patient-years).^20^ Our findings in older age groups are also consistent with cardiovascular disease overtaking cancer as the leading cause of death in the general population in older age groups.

We also previously showed that patterns of cardiovascular risk varied among cancer sites and by receipt of chemotherapy. The causes of increases in cardiovascular mortality in cancer survivors over time may therefore relate to differences in treatment among cancer sites, as well as general risk factors such as age that are shared with the general population. Differences in crossover points among cancer sites are also driven by different patterns in mortality due to the primary cancer over time. For example, mortality due to bladder and uterine cancer drops steeply early in survivorship, partly explaining early crossover points for these cancer sites.

### Study strengths and limitations

A strength of our study is the use of large-scale linked data that are representative of the English population with high validity (positive predictive value and sensitivity) for identifying cancer.^21^ Selection bias due to false and missing cancer diagnoses will therefore be minimal. Cause-specific mortality was identified using death registration data that are collected following internationally agreed rules and relies on the quality of assignment of underlying cause of death by clinicians.^22^ These data were available for all English residents until the end of the coverage period of the study. Observed loss to follow-up was therefore due to death or the end of the coverage period, which restricted follow-up to 20 years; this prevented exploration of cause-specific mortality over longer time periods, which would be likely to lead to the observation of crossover points in the younger age groups due to normal processes of aging potentially combined with delayed effects of cancer treatments on cardiovascular health. The only mechanism of loss to follow-up leading to missing death data was moving to another country before the end of the study period; we assume that this would be minimal.

Post hoc sensitivity analysis showed minimal differences when using the immediate rather than underlying cause of death in our outcome definition. A key limitation of our study is that we had insufficient data to stratify by cancer characteristics (eg, stage, grade) and treatment (eg, receipt of known cardiotoxic drugs or radiation therapy to the heart). In addition, for some cancers, CIs for our estimated crossover points between cardiovascular and cancer mortality were wide because of limited power. We included only people older than 40 years because of limited power in younger age groups, so the impact of any increased cardiovascular risks in middle age among people diagnosed with cancer as children or young adults could not be investigated.

Despite advances in the treatment of cancer, research continues to demonstrate that the most likely cause of death after cancer diagnosis is the primary cancer itself,^4^^,^^23^, ^24^, ^25^, ^26^, ^27^ because of high cancer mortality rates in the early years following cancer diagnosis. Postcancer monitoring and care has tended to focus on managing risk for cancer relapse, but we have shown that in older people, as time passes since cancer diagnosis, the risk for cardiovascular disease mortality overtakes a declining risk for cancer mortality. This highlights the importance of developing strategies to monitor and prevent long-term cardiovascular risk in cancer survivors. Future research identifying specific groups of cancer survivors who are at increased risk for cardiovascular disease (eg, by treatment group or tumor characteristics) would help target these measures.

## Conclusions

Our observations of a substantial cardiovascular mortality risk in older cancer survivors, which overtakes cancer mortality for survivors of several cancers in older age groups several years after cancer diagnosis, strengthen calls to raise awareness of cardiovascular disease risk in older cancer survivors and to prioritize the prevention of cardiovascular disease alongside the prevention of cancer recurrence as time passes since cancer diagnosis. Cancer mortality rates exceeded cardiovascular mortality over the follow-up period for most cancers in the younger age group (40-59 years) despite previously observed relative increases in cardiovascular disease in this age group compared with the general population.

Perspectives **COMPETENCY IN MEDICAL KNOWLEDGE:** In older survivors of 9 common cancers, cardiovascular mortality becomes dominant over mortality from the primary cancer, though not always over total cancer mortality, as time passes since cancer diagnosis.

**TRANSLATIONAL OUTLOOK:** These findings highlight the importance of prioritizing monitoring of both cancer recurrence and cardiovascular disease in older cancer survivors to the cardiovascular, oncology, and primary care communities. Future studies should determine the effectiveness of cardiovascular risk prediction and prevention methods in cancer survivors.

## Funding Support and Author Disclosures

This work was supported by the Wellcome Trust and the Royal Society (grants 107731/Z/15/Z and 220283/Z/20/Z). Dr Lyon is supported by the Fondation Leducq Network of Excellence in Cardio-Oncology. Dr Lyon has received grants and personal fees from Servier and Pfizer and personal fees from Novartis, Roche, Takeda, Boehringer Ingelheim, Amgen, Clinigen Group, Ferring Pharmaceuticals, Eli Lily, Bristol Myers Squibb, and Eisai, outside the submitted work. Dr Bhaskaran has received grants from the Wellcome Trust and the Royal Society during the conduct of the study; and has received grants from the Medical Research Council, the British Heart Foundation, and Diabetes UK, outside the submitted work. Dr Smeeth has received grants from the Wellcome Trust, the Medical Research Council, the National Institute for Health Research, GlaxoSmithKline, the British Heart Foundation, and Diabetes UK, outside the submitted work; and is a trustee of the British Heart Foundation. Dr Stanway has received personal fees from Roche, Novartis, Eli Lilly, and Clinigen, outside the submitted work. Dr dos-Santos-Silva has received grants from Susan G. Komen and the National Cancer Institute, outside the submitted work. Dr Matthews has received grants from Forte, outside the submitted work. All other authors have reported that they have no relationships relevant to the contents of this paper to disclose.
