# Supplementary material for: Does Cardiovascular Mortality Overtake Cancer Mortality During Cancer Survivorship? An English Retrospective Cohort Study
Source: JACC CardioOncol. 2022 Mar 15;4(1):113–23. doi: 10.1016/j.jaccao.2022.01.102 (PMC9040113; doi:10.1016/j.jaccao.2022.01.102)

**Supplemental Appendix**

Predicted cause-specific mortality per 1000 person-years by time since diagnosis in cancer survivors

1. Males and females
2. Immediate cause of death (in place of underlying cause of death)
3. All malignancy (in place of primary malignancy)

____ Cardiovascular disease mortality _ _ _ Primary malignancy mortality ……. All malignancy mortality

Intersect = the time point in years when cardiovascular mortality rate equals primary (or all) malignancy mortality rate. Ordering of cancer sites is by ICD code of the cancer site. Graphs are truncated at the earliest time since diagnosis group where the width of the confidence intervals exceeds 50/1000 patient years.

**Supplemental Figure 1A** (male, 40-59 yrs)


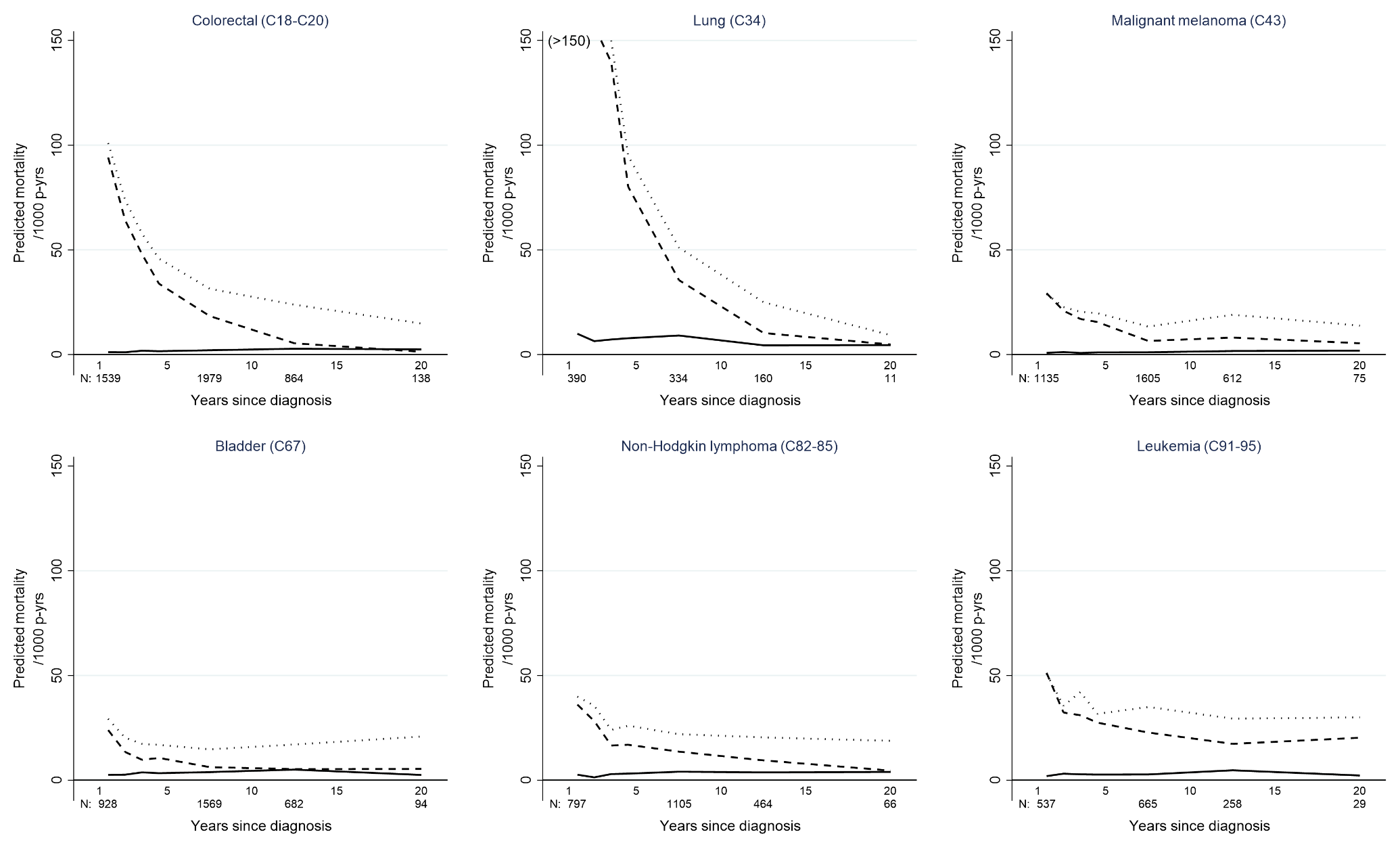


**Supplemental Figure 1B** (female, 40-59 years)


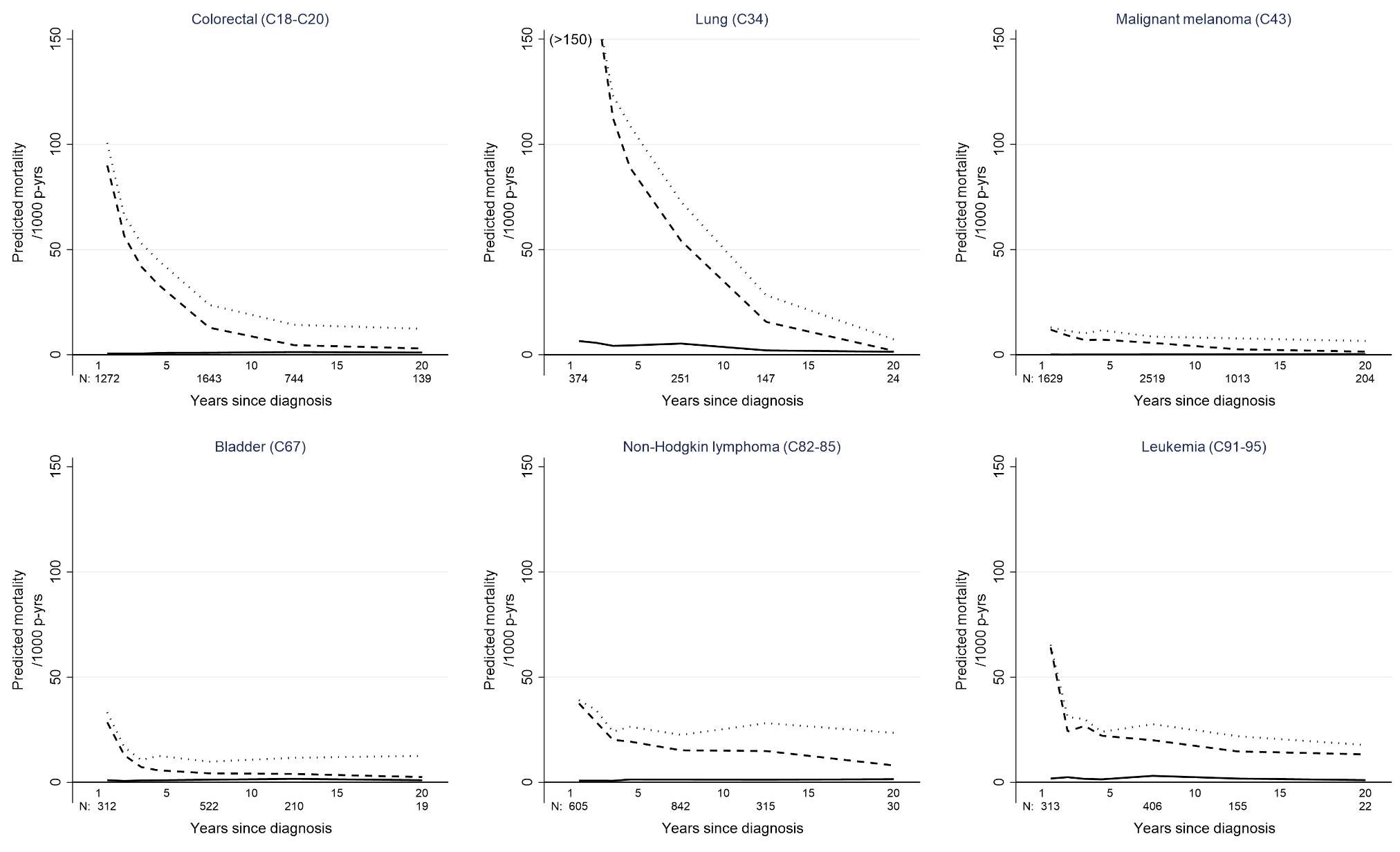


**Supplemental Figure 1C** (male, 60-79 years)


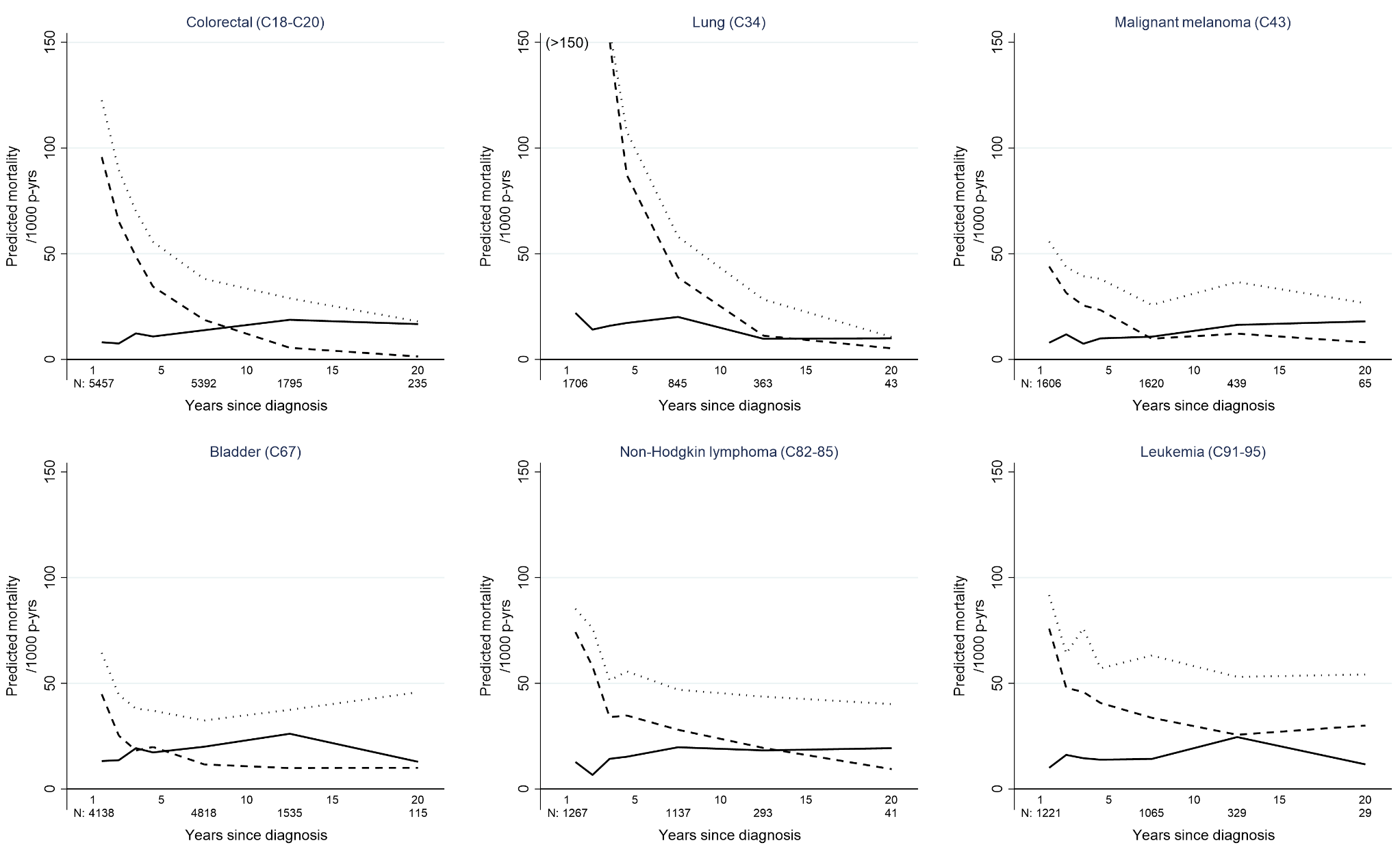


**Supplemental Figure 1D** (female, 60-79 years)


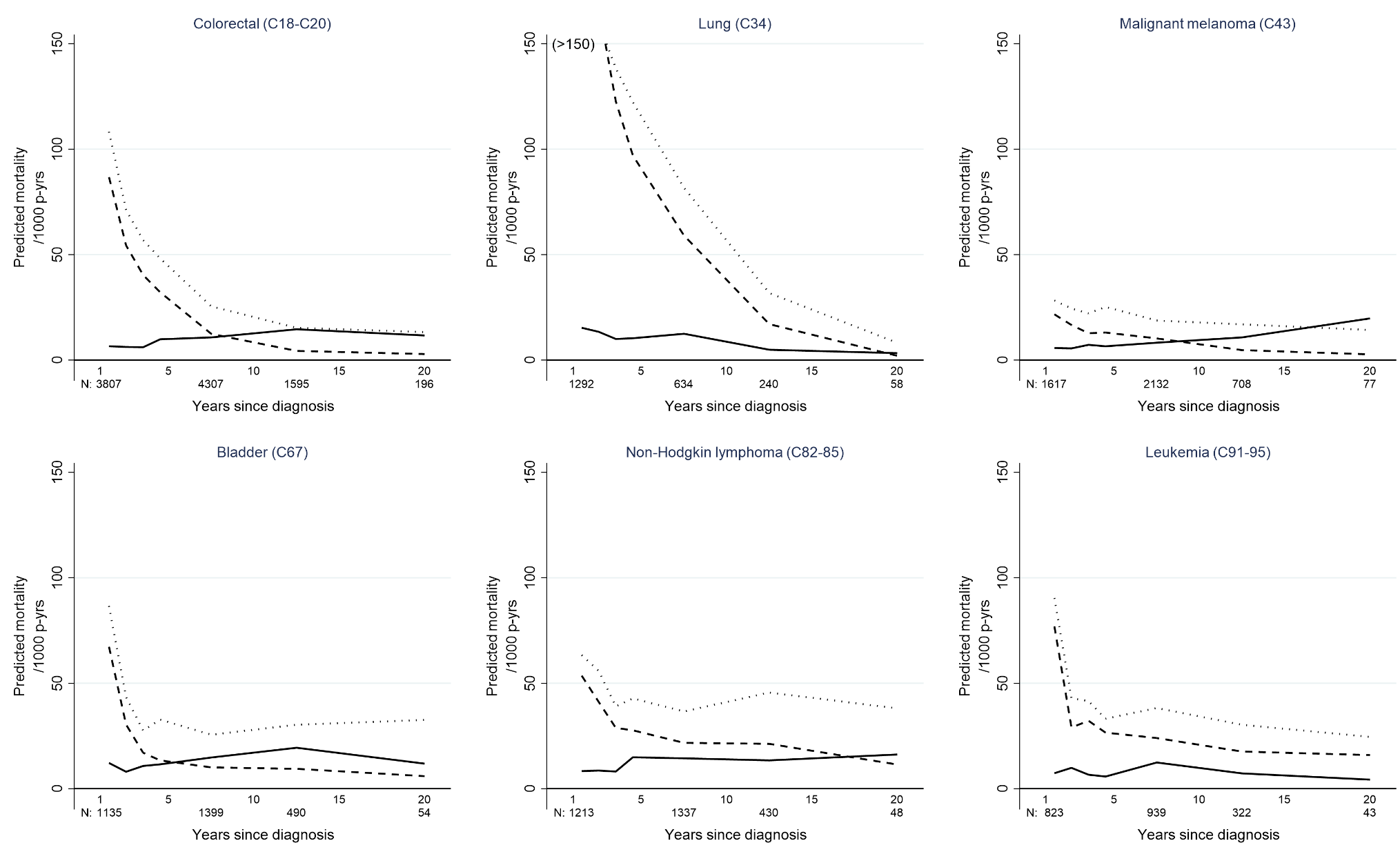


**Supplemental Figure 1E** (male, ≥80 years)


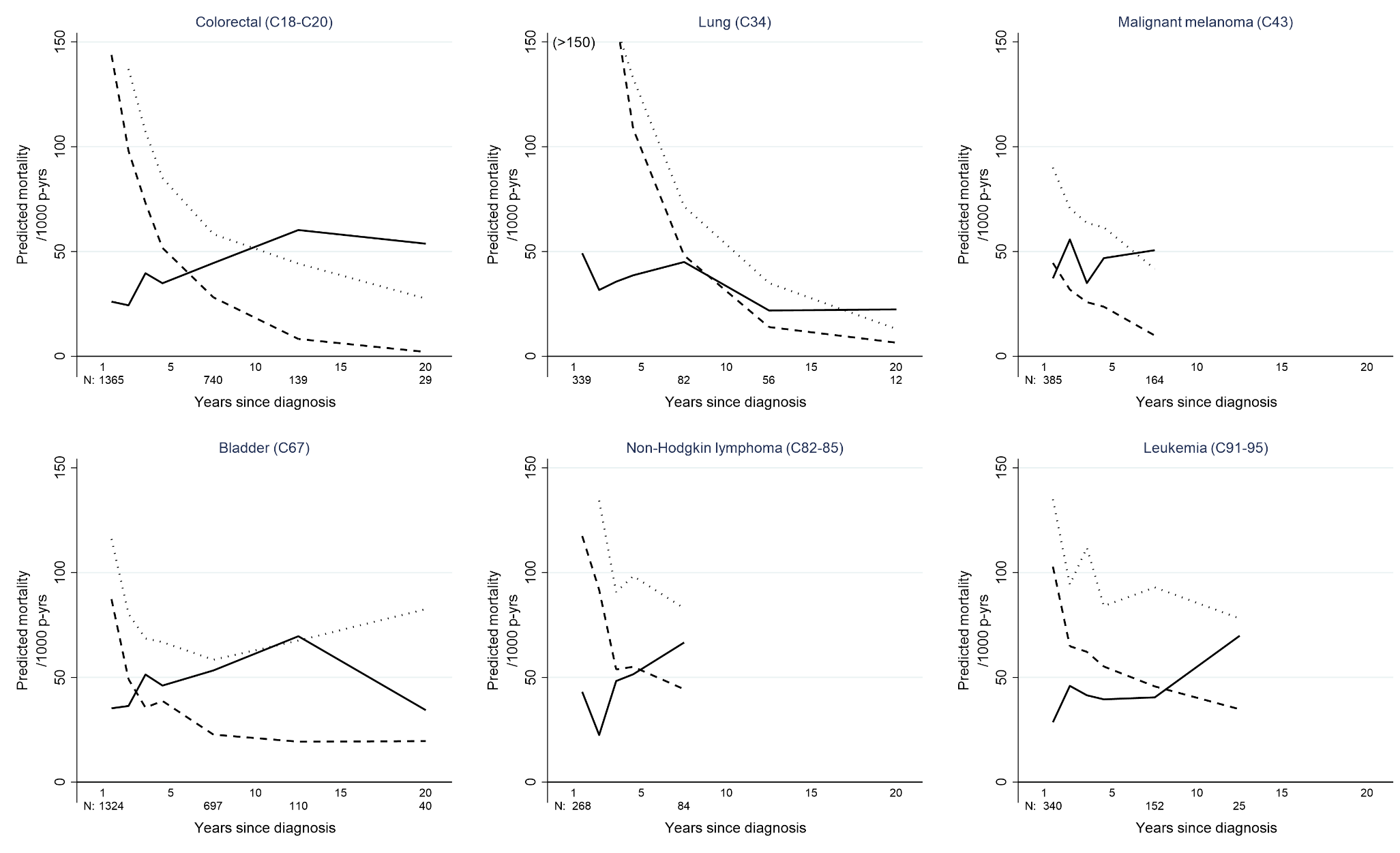


**Supplemental Figure 1F** (female, ≥80 years)


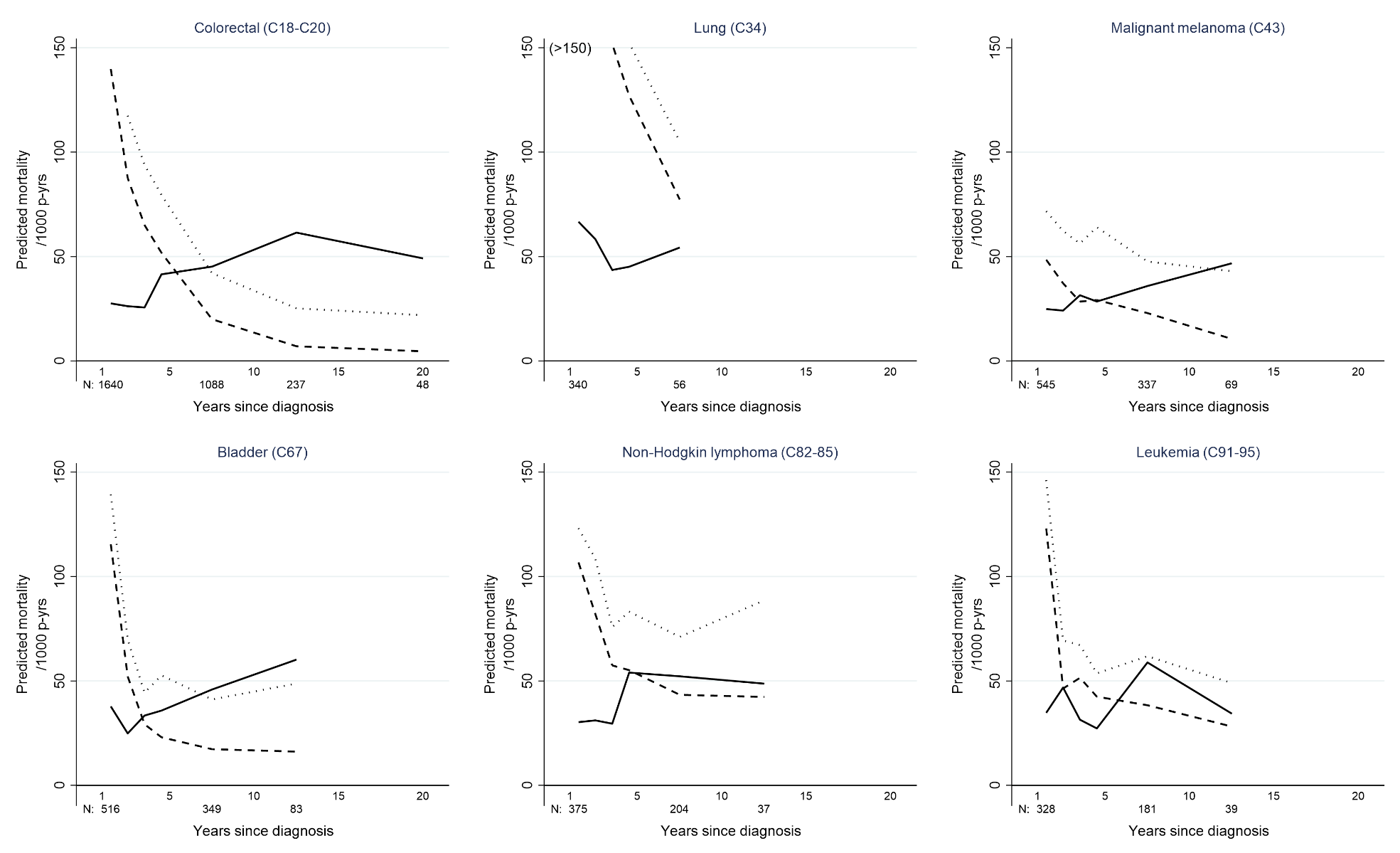


**Supplemental Figure 2A** (immediate cause of death, 40-59 years)


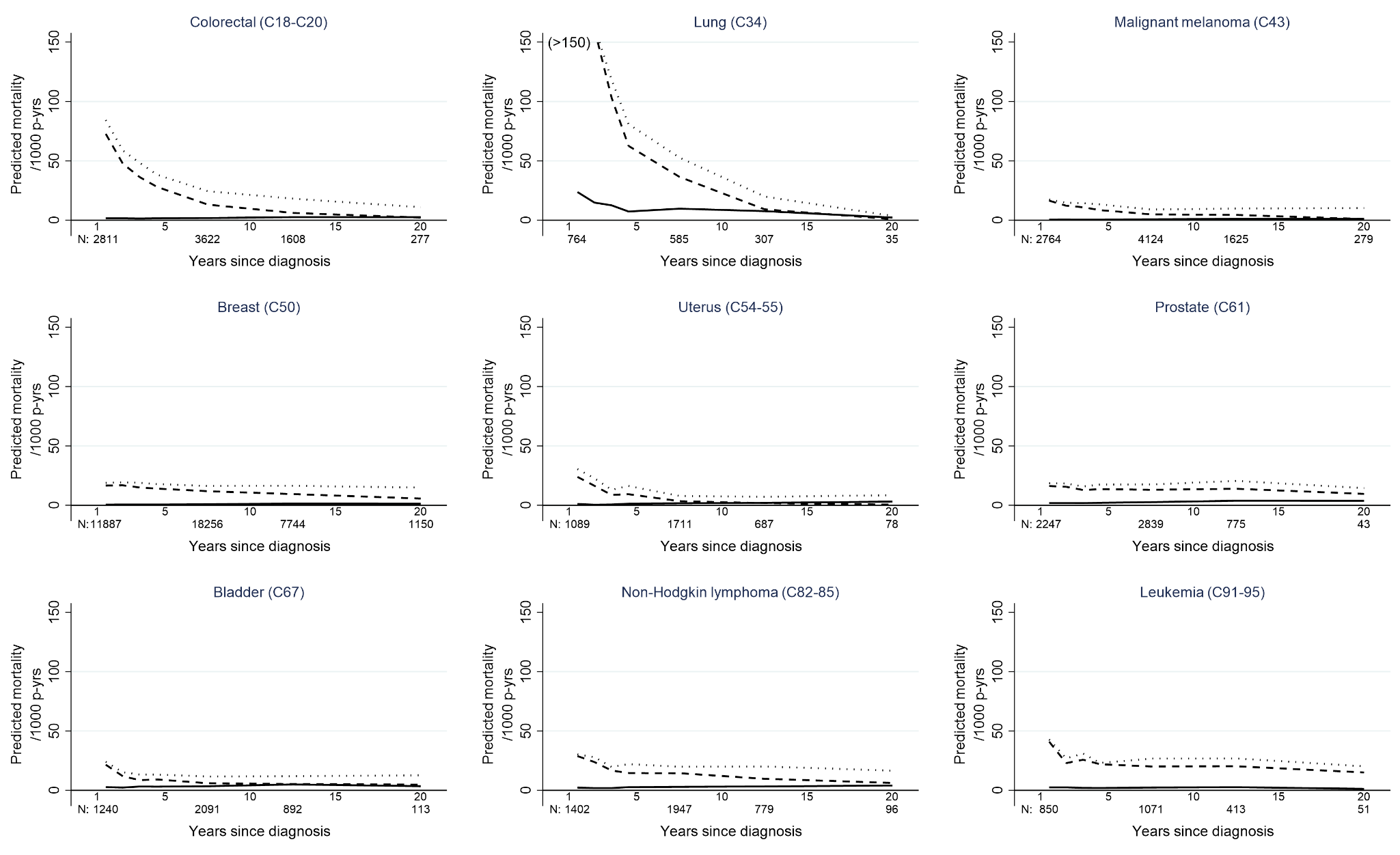


**Supplemental Figure 2B** (immediate cause of death, 60-79 years)


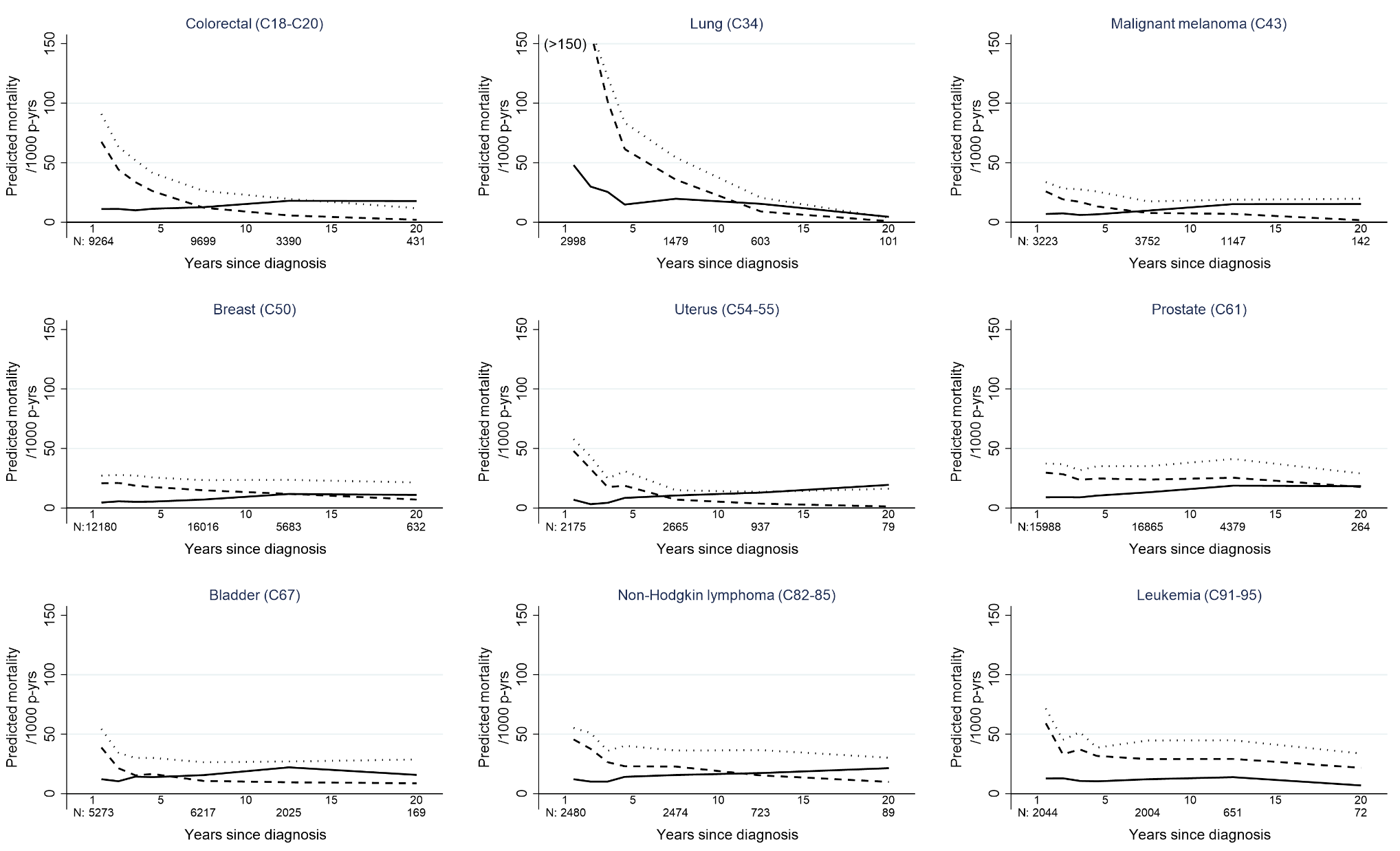


**Supplemental Figure 2C** (immediate cause of death, ≥80 years)


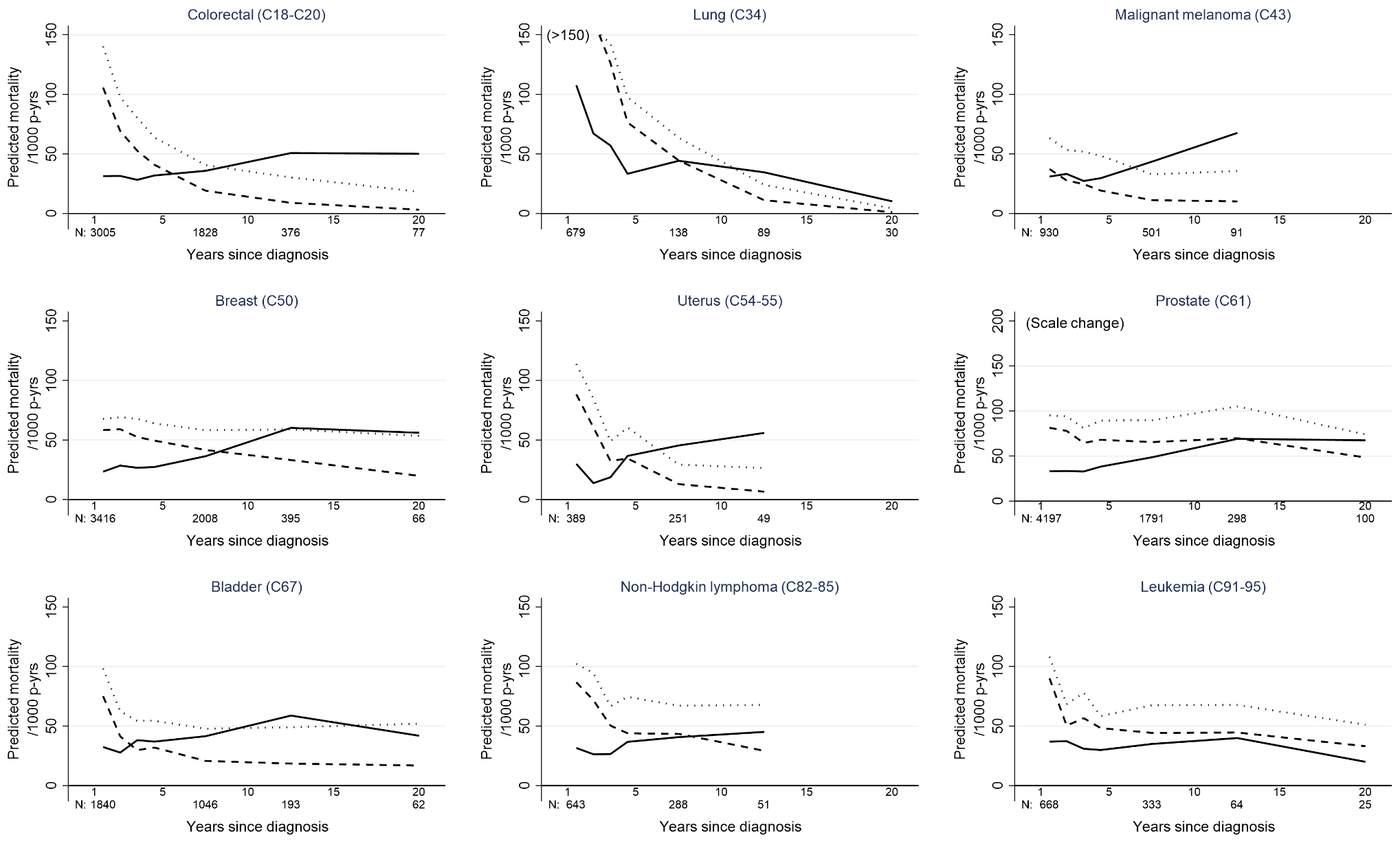

Supplement: Supplemental Data [file mmc1.docx]
